# Highly active antiretroviral therapy for critically ill HIV patients: A systematic review and meta-analysis

**DOI:** 10.1371/journal.pone.0186968

**Published:** 2017-10-24

**Authors:** Hugo Boechat Andrade, Cassia Righy Shinotsuka, Ivan Rocha Ferreira da Silva, Camila Sunaitis Donini, Ho Yeh Li, Frederico Bruzzi de Carvalho, Pedro Emmanuel Alvarenga Americano do Brasil, Fernando Augusto Bozza, Andre Miguel Japiassu

**Affiliations:** 1 Intensive Care Unit of Instituto Nacional de Infectologia Evandro Chagas, Fundação Oswaldo Cruz (Fiocruz). Rio de Janeiro, RJ, Brazil; 2 Infectious Diseases Intensive Care Unit of Hospital das Clínicas da Faculdade de Medicina da Universidade de São Paulo. São Paulo, SP, Brazil; 3 Intensive Care Unit of Hospital Eduardo de Menezes da Fundação Hospitalar do Estado de Minas Gerais. Belo Horizonte, MG, Brazil; Universidade do Extremo Sul Catarinense, BRAZIL

## Abstract

**Introduction:**

It is unclear whether the treatment of an HIV infection with highly active antiretroviral therapy (HAART) affects intensive care unit (ICU) outcomes. In this paper, we report the results of a systematic review and meta-analysis performed to summarize the effects of HAART on the prognosis of critically ill HIV positive patients.

**Materials and methods:**

A bibliographic search was performed in 3 databases (PubMed, Web of Science and Scopus) to identify articles that investigated the use of HAART during ICU admissions for short- and long-term mortality or survival. Eligible articles were selected in a staged process and were independently assessed by two investigators. The methodological quality of the selected articles was evaluated using the *Methodological Index for Non-Randomized Studies* (MINORS) tool.

**Results:**

Twelve articles met the systematic review inclusion criteria and examined short-term mortality. Six of them also examined long-term mortality (≥90 days) after ICU discharge. The short-term mortality meta-analysis showed a significant beneficial effect of initiating or maintaining HAART during the ICU stay (random effects odds ratio 0.53, p = 0.02). The data analysis of long-term outcomes also suggested a reduced mortality when HAART was used, but the effect of HAART on long-term mortality of HIV positive critically ill patients remains uncertain.

**Conclusions:**

This meta-analysis suggests improved survival rates for HIV positive patients who were treated with HAART during their ICU admission.

## Introduction

The epidemiology of human immunodeficiency virus (HIV) infection and acquired immune deficiency syndrome (AIDS) has changed since the introduction of highly active antiretroviral therapy (HAART) in 1996 [1,2]. The mortality attributable to AIDS-related opportunistic infections has decreased significantly, and a growing proportion of treatment adherent HIV-infected patients are now living with controlled HIV replication and improved immune function [3–5]. The observed increased survival has led to aging and a higher prevalence of chronic diseases in this population, such as cardiovascular and pulmonary diseases, malignancies and even a unique set of comorbidities, which are now designated as HIV-associated non-AIDS (HANA) conditions [6]

HIV-infected people are at higher risk for critical illnesses than demographically matched HIV-negative individuals with similar comorbidities [6]. Opportunistic infections are still the leading cause of intensive care admissions, mainly in low- and middle-income countries or among patients with low treatment adherence or low healthcare access [7,8]. On the other hand, HANA conditions have gained increasing importance as causes for intensive care unit (ICU) admissions [6,9]. Recent evidence suggests that short-term ICU mortality has decreased in HIV-infected patients, independently of CD4+ cell counts, HIV load or previous HAART use, which is likely due to increased access and improved critical care management in the last decade [9].

However, it is still uncertain which aspects of the clinical management of HIV affect the patient’s outcome in the ICU. The use of HAART in this setting, as initiation or maintenance, is still a matter of concern, due to possible changes in its bioavailability, associated adverse effects, and the risk of induction of viral resistance [7,8,10].

Therefore, we performed a systematic review and meta-analysis of published studies that focused on the impact of HAART on the outcomes of HIV-infected patients admitted to the ICU. To our knowledge, a systematic review on the use of HAART during the ICU stay has not been performed so far. Consequently, our hypothesis was that the use of HAART during critical illness could influence the short-term (ICU mortality and in-hospital mortality after ICU discharge) and/or the long-term (mortality ≥ 90 days after ICU discharge) outcomes.

## Materials and methods

This study was registered on the International Prospective Register of Systematic Reviews (PROSPERO http://www.crd.york.ac.uk/PROSPERO/): registration number CRD42015026739.

An electronic search was performed on 09/14/2017 using three online medical reference databases: PubMed (MEDLINE http://www.ncbi.nlm.nih.gov/pubmed/), Web of Science (Thomson Reuters http://wokinfo.com/) and Scopus (Elsevier http://www.scopus.com/) to identify studies published in English from the time that HAART was considered a viable treatment option and that investigated the prognostic factors for survival or mortality in critically ill adult patients with HIV/AIDS. The search was conducted for short-term and long-term mortalities.

The following search string was used on PubMed and Web of Science: ("HIV" OR "Acquired Immunodeficiency Syndrome Virus" OR "AIDS Virus" OR "AIDS Viruses" OR "Virus, AIDS" OR "Viruses, AIDS" OR "Acquired Immunodeficiency Syndrome" OR "Immunologic Deficiency Syndrome, Acquired" OR "Acquired Immune Deficiency Syndrome" OR "Acquired Immunodeficiency Syndrome") AND ("Critical Care"[Mesh] OR "Intensive Care" OR "Critical Illness" OR "Critically Ill" OR "Intensive Care Units").

Specific string terms were used for Scopus due to its search engine characteristics: search—TITLE-ABS-KEY (hiv) OR TITLE-ABS-KEY (aids) AND TITLE-ABS-KEY (critical care) OR TITLE-ABS-KEY (intensive care) OR TITLE-ABS-KEY (critically ill) OR TITLE-ABS-KEY (critical illness) AND SUBJAREA (mult OR bioc OR immu OR neur orphar OR mult OR medi OR nurs OR heal) AND PUBYEAR > 1995.

The articles were identified in a staged process whereby titles were initially screened for potential eligibility by a single reviewer (HBA). Exclusion criteria were as follow: review articles, letters to the editor, opinions, or comments; articles that evaluated pregnant women or pediatric populations; articles that lacked HIV-or ICU-specific data; and articles that lacked an outcome evaluation or had no outcome of interest (mortality/survival).

Following this selective review, the abstracts were read by two reviewers (HBA and CRS) for possible eligibility. Articles were eligible if the following criteria were met: publication date after 01/01/1996; study of adult humans (age>18 years); information collected after HAART was approved for use; risk factors for the outcomes of interest; and clear and available information about the outcome of interest: short-term mortality (ICU mortality, or hospital mortality after ICU discharge), or long-term (≥90 days) mortality after ICU discharge.

Finally, the reviewers (HBA and CRS) searched full-text articles that focused specifically on the impact of the use of HAART during the ICU stay and/or the prognosis of critically ill HIV patients and included articles that reported short- and long-term mortality data in regression analyses. The bibliographies of the articles were also reviewed to determine if any relevant studies could be considered. Disagreements between reviewers were resolved by a third reviewer (AMJ).

Data was extracted onto standardized data extraction REDCap (web-based data capture tool for research studies www.project-redcap.org) sheets independently by HBA and CRS (data extraction sheets available on request). The following data were collected from each article: study design, method of data collection, number of HIV patients, mortality with and without HAART and demonstrated strength of association (e.g., data gathered on the odds (OR) and/or hazard (HR) ratios, 95% confidence intervals (95%CI), and p-values (<0.05) for each of the studied outcomes). The methodological quality of reporting of the selected articles was assessed by two reviewers (HBA and CRS) using the *Methodological Index for Non-Randomized Studies* (MINORS) tool score system [11].

The software used for data analysis was the Review Manager 5.3.5 for Mac OS X (2017—http://tech.cochrane.org/revman/download) after importing REDCap data as Microsoft Excel 2016 compatible spreadsheets files. The comparison of the dichotomous primary outcome (death; yes/no) was conducted using a conventional Mantel-Haenszel random effects meta-analysis and posterior sensitivity analyses, which were reported as odds ratios with 95% confidence intervals (CI). Heterogeneity of study-specific results was quantified using the descriptive I^2^ inconsistency measure. Possible publication bias was inspected using asymmetry tests (funnel plots).

ICU and hospital mortalities were combined as short-term mortality. When an article presented both ICU and hospital mortalities data, the latter was used as outcome [12]. We also performed sensitivity analyses to check for the effects of possible heterogeneity among published articles.

## Results

**Fig 1:**
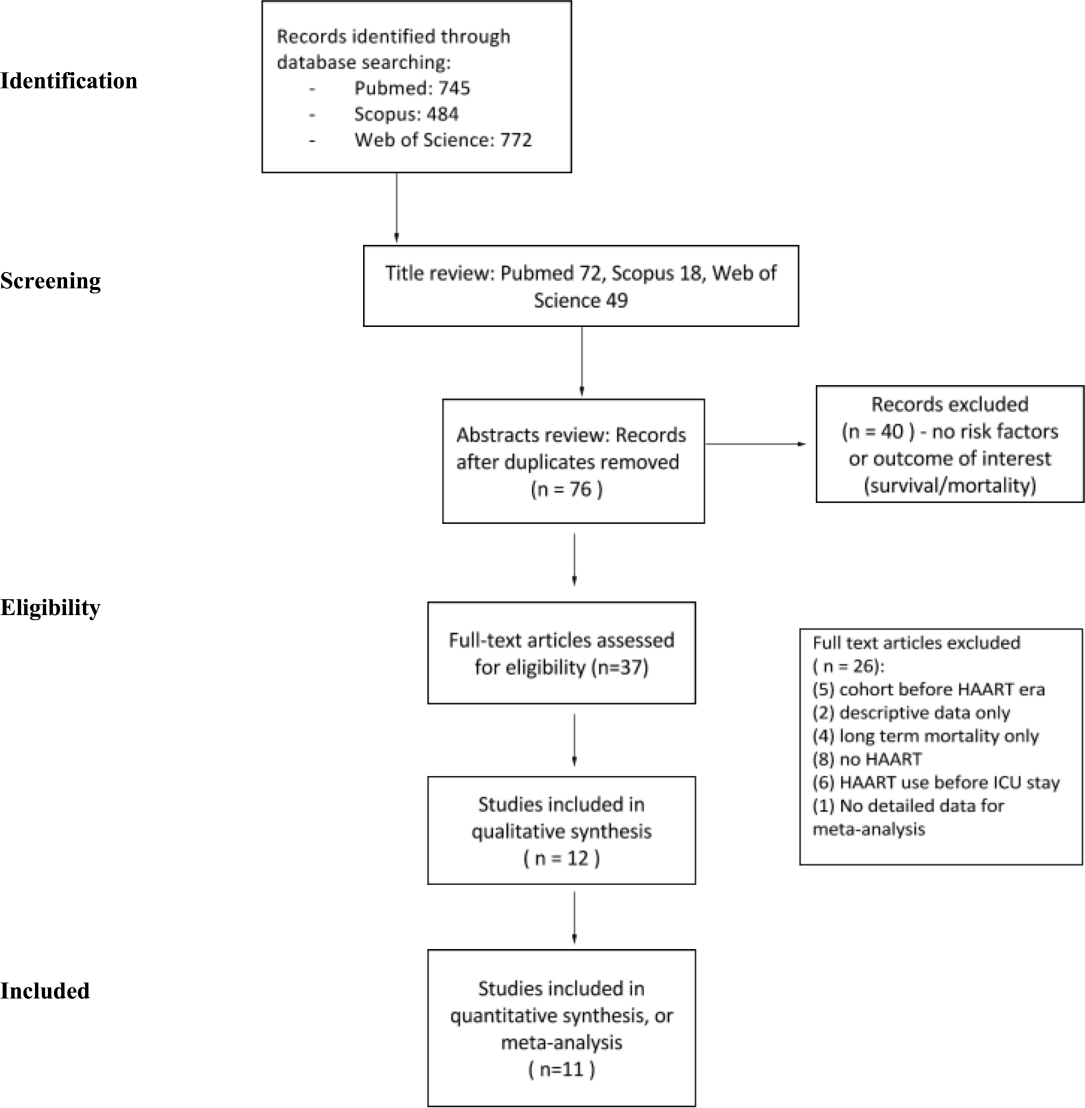
Highly active antiretroviral therapy systematic review and meta-analysis flowchart. HAART systematic review flowchart, which shows the procedure used for the selection of the articles for the qualitative and quantitative synthesis. *HAART* highly active antiretroviral therapy; *ICU* intensive care unit. PRISMA Model Flow Diagram [24].

In the first step, 745 articles from PubMed, 772 from Web of Science and 484 from Scopus were identified. After title review, the original results were filtered to 72 studies from PubMed, 49 from Web of Science and 18 from Scopus. Duplicates were excluded, which resulted in 76 abstracts that were potentially eligible. After further screening, 37 full-text articles and their references were assessed. Finally, 12 papers [7,13–23] were included in the qualitative synthesis, for systematic review. Chiang *et al*. showed no detailed data for the quantitative synthesis, so the remaining eleven were meta-analyzed for short-term mortality (ICU mortality and hospital mortality after ICU discharge–Table A in S1 File). Fig 1 represents the review flowchart.

The selected 12 articles were conducted in various countries and were published between 2003 and 2015. All the articles were single center retrospective cohort studies, with an absolute total number of 1,584 HIV patients who were admitted for intensive care. Generally, the characteristics of the population in most studies were similar. The mean CD4 cell count was below 200 cells/mm^3^ in all studies. Respiratory failure was the most frequent cause of ICU admission in 10 of 12 studies. Nine out of the 12 studies included all HIV patients admitted to the ICU, and 3 studies selected a subset of HIV patients (sepsis, *Pneumocystis jiroveci* pneumonia, or acute respiratory failure). Conversely, the length of follow-up was diverse among the selected studies: 5 studies followed patients up to hospital discharge, 2 studies followed patients for 6 months, another 2 studies followed patients for a year, and 3 other studies presented different periods of follow-up. Table 1 summarizes the general characteristics of the studies.

**Table 1:** General characteristics of the studies.

Author	Country	Period	Study Design	CD4 (cells/mm^3^)	Follow-up	Severity	Main cause of admission	Inclusion criteria
**Adlakha**	London, UK	Jan99-Jun09	Retrospective cohort	110 (20–340)	Hospital discharge	APACHE II 16.5 (13–23)	PCP (50%)	HIV-infected patients
**Amâncio**	Belo Horizonte, Brazil	Jan-Dec06	Retrospective cohort	116 (±172)	24 months	APACHE II 22 (12)	Respiratory failure (43.20%)	HIV-infected patients
**Barbier**	Paris, France	Jan96-Dec06	Retrospective cohort	192 (46–393)	Hospital discharge	SOFA 4 (3–7)	Respiratory failure (100%)	HIV-infected patients with acute respiratory failure
**Chiang**	Taipei, Taiwan	Jan01-Feb10	Retrospective cohort	30 (13–103)	100 days	APACHE II 19 (15–25)	Respiratory failure (78.5%)	HIV-infected patients
**Cribbs**	Atlanta, USA	2006–2010	Retrospective analysis of a prospective cohort	41 (8–161)	Hospital discharge	APACHE II 26.3±7.9	Sepsis (100%)	All septic HIV patients from a prospective cohort
**Croda**	São Paulo, Brazil	Oct96-Oct06	Retrospective cohort	39 (16–92)	6 months	APACHE II 19	Respiratory failure (33%) and sepsis (31.3%)	HIV-infected patients >24 h
**Greenberg**	Atlanta, USA	Oct06-Jan09	Retrospective cohort	30 (1–1501)	Hospital discharge	APACHE II 24 ±5	Sepsis (100%)	HIV-infected patients with sepsis
**Meybeck**	Tourcoing, France	Jan00-Dec09	Retrospective cohort	112 (1–935)	6 months	SAPS II 47 ± 20	Respiratory failure (51%)	HIV-infected patients
**Morquin**	MontpellierFrance	Jan97-Dec08	Retrospective cohort	173.5 ± 192	1 year	SAPS II 53.8 ± 20.7	Respiratory failure (38.8%)	HIV-infected patients
**Morris**	San Francisco, USA	Jan96-Jun01	Retrospective cohort	19 (1–580)	Hospital discharge	APACHE II 13 (4.5)	Respiratory failure (100%)	HIV-infected patients with confirmed severe PCP
**van Lelyveld**	Utrecht, The Netherlands	Jul06-Dec08	Retrospective cohort	83 (0–642)	5 years	APACHE II 25 (10–41)	Respiratory failure (43%)	HIV-infected patients, excluding postoperative patients and intoxications
**Vargas—Infante**	Mexico City, Mexico	Dec85-Jan06 Nov96-Jan06 HAART era	Retrospective cohort	Aids CDC criteria 90.5%	5 years	APACHE II 11 (2–26) HAART era	Respiratory failure (86%) HAART era	HIV-infected patients

*APACHE II* Acute Physiology and Chronic Health Evaluation II score; *SAPS II* Simplified Acute Physiology Score II; *SOFA* Sequential Organ Failure Assessment score; *PCP Pneumocystis jiroveci* pneumonia; *HAART* highly active antiretroviral therapy

The methodological quality of the 12 studies were heterogeneous. Table 2 compares the methodological quality of the selected studies using the MINORS score system.

**Table 2:** Comparison of the methodological quality of articles using the *Methodological Index for Non-Randomized Studies (MINORS)* score system [11].

Criteria	Adlakha	Amâncio	Barbier	Chiang	Cribbs	Croda	Greenberg	Meybeck	Morquin	Morris	van Lelyveld	Vargas Infante
**1**	1	2	2	2	2	2	2	2	2	2	2	2
**2**	2	2	1	2	2	2	0	2	2	2	2	2
**3**	1	1	1	1	1	1	1	1	1	1	1	1
**4**	1	2	2	2	2	2	1	2	1	2	2	2
**5**	0	0	0	0	0	0	0	0	0	0	0	0
**6**	2	2	2	2	2	2	2	2	2	2	2	1
**7**	2	0	2	2	2	1	2	1	1	2	1	2
**8**	0	0	0	0	0	0	0	0	0	0	0	0
**9**	1	0	2	1	2	2	0	2	1	2	2	2
**10**	1	0	1	2	2	2	0	2	2	2	1	1
**11**	1	0	1	2	2	2	0	2	1	2	2	2
**12**	2	0	1	1	2	2	0	1	2	1	0	2
**Total** **score**	**14**	**9**	**15**	**17**	**19**	**18**	**8**	**17**	**15**	**18**	**15**	**17**

The MINORS criteria are: 1. An openly stated aim; 2. Inclusion of consecutive patients; 3. Prospective collection of data: data were collected according to a protocol established before the beginning of the study; 4. Endpoints appropriate to the aim of the study; 5. Unbiased assessment of the study endpoint; 6. Follow-up period appropriate to the aim of the study; 7. Loss to follow-up of less than 5%; 8. Prospective calculation of the study size; 9. An adequate control group; 10. Contemporary groups; 11. Baseline equivalence of groups; 12. Adequate statistical analysis. The items are scored 0 (not reported), 1 (reported but inadequate) or 2 (reported and adequate).

Although all studies were retrospective in nature and the main goals were stated, there was bias in the assessment of endpoints, loss of follow-up was common (5 out of 12 studies), and the control group was not equivalent or contemporary to the studied group (6 out of 12). No study reported calculation of the its sample size. The twelve criteria used for scoring are detailed on the Appendix in S1 File.

### Effects on short-term mortality

**Fig 2:**
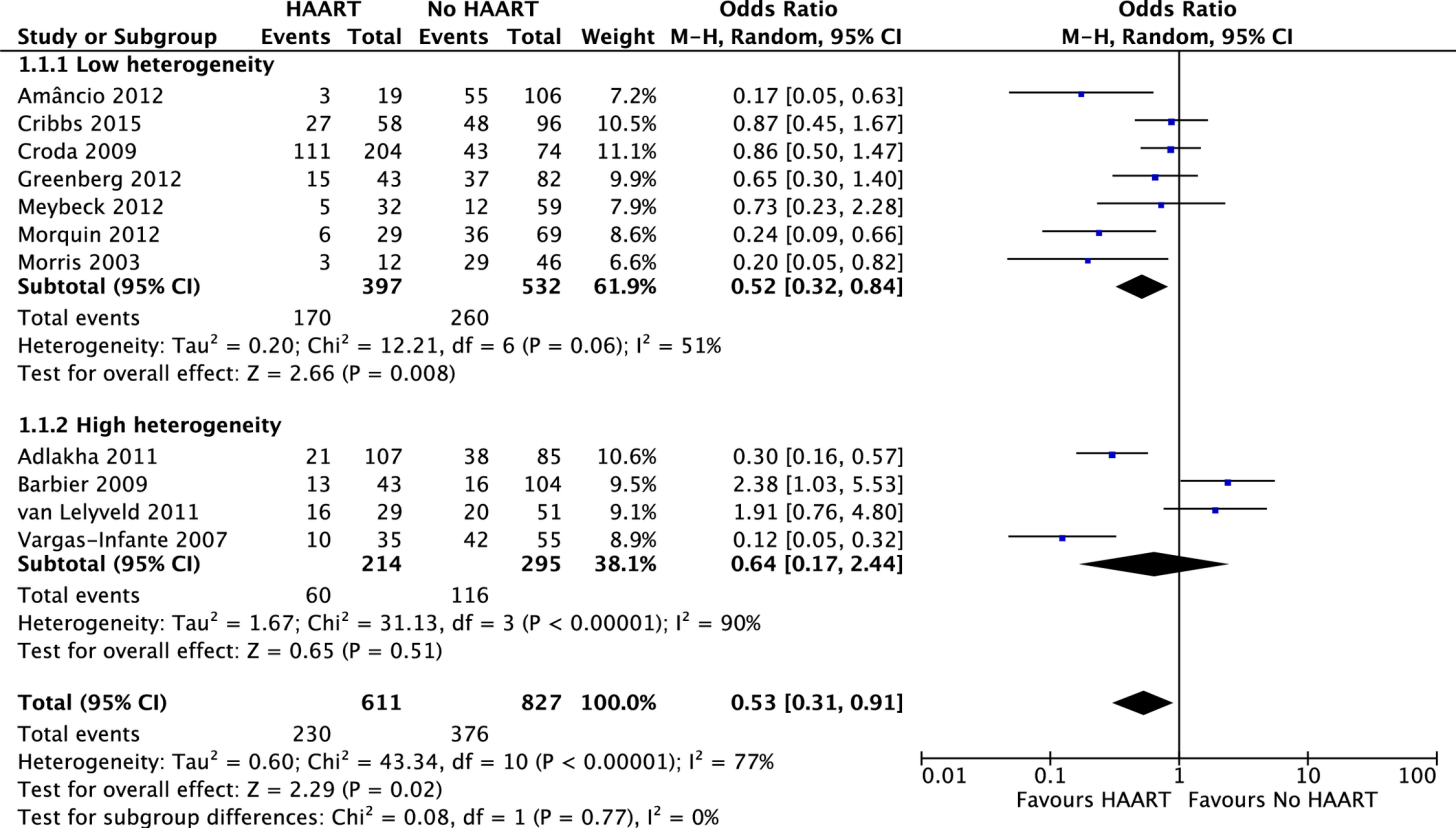
Forest plot of the effects of highly active antiretroviral therapy (HAART) on *short-term* mortality–random effects odds ratio.

**Fig 3:**
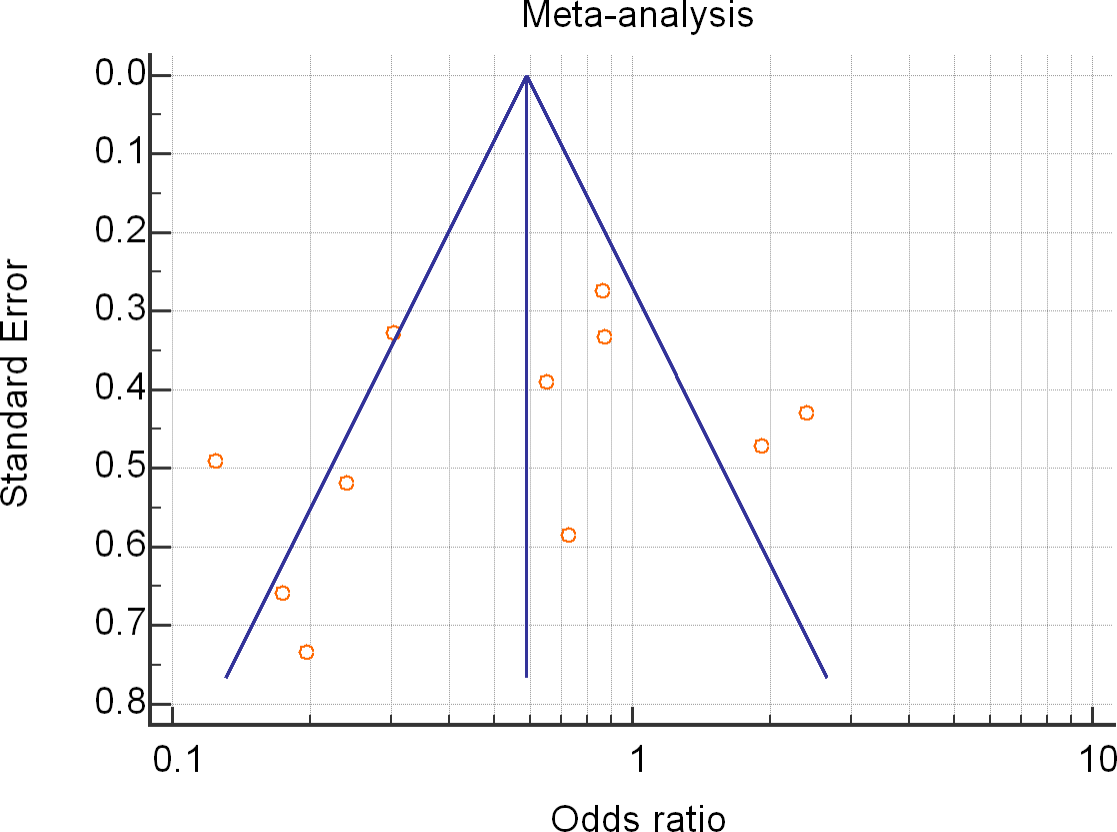
Funnel plot of the effects of highly active antiretroviral therapy (HAART) on *short-term* mortality–random effects odds ratio.

The meta-analysis of eleven articles [7,13–15,17–23] that reported short-term mortality confirmed the reduction of short-term mortality in the HAART-exposed group with a random effects odds ratio of 0.53, 95%CI 0.31–0.91, z-score -2.29, p = 0.02. However, the heterogeneity was high (I^2^ 77%) with a significance level of p<0.00001. The funnel plot analysis showed four studies [13,15,21,22] outside the 95%CI region. Fig 2 shows the forest plot and Fig 3 the funnel plot of HAART random effects model on short-term mortality.

We investigated whether the initiation or maintenance of HAART during the ICU stay were associated with the outcome. The information on HAART prescription during the ICU stay was not uniform across all articles. It was reported in only 6 articles [7,17,19–21,23], while it was not informed in the remaining 5 articles. Overall, there was no observed difference in outcome whether HAART was maintained or initiated during the ICU stay.

### Effects on long-term mortality after ICU discharge

Six articles [7,14,18,19,21,22] evaluated long-term follow-up (≥90 days) after ICU discharge (Table A in S1 File). Three of them presented statistically significant beneficial effects of the use of HAART during the ICU stay after 6 months [7], 1 year [19] and 5 years [22]. The other three studies [14,18,21] showed no benefit. Even so, it was not possible to meta-analyze them due to lack of report of specific data on HAART. A Pearson's chi-square test with a p-value>0.05 suggested no association with the outcome, aborting initial plans on performing a meta-regression.

## Discussion

This systematic review and meta-analysis is a comprehensive assessment of the possible effect of the use of HAART, during ICU stay, in critically ill HIV positive patients. It provides data from a total of 1,584 critically ill HIV positive patients from 12 studies, all of which were retrospective in nature and presented roughly similar population characteristics. Our data highlights a reduction in short-term mortality with the use of antiretroviral treatment during the ICU stay. There was also an observed trend for lower long-term mortality with the use of HAART in this setting. However, we noticed a wide heterogeneity of methodological quality among the studies, which precludes a stronger assertion about the protective effects of HAART for critically ill HIV patients.

Prescribing HAART in the ICU setting presents distinct challenges related to drug interactions [10], doses and delivery [25], as well as antiretroviral-associated toxic effects [26]. As few studies are currently available to guide the use of HAART in critically ill HIV-infected patients, the decision to initiate or maintain HAART in this situation is subjective, as there is no current consensus among experts [27]. Selection bias might be an issue as the sickest patients, who likely have a higher risk of death, would not be perfect candidates for the use of antiretroviral drugs due to their clinical status, especially in patients with shock syndromes and/or possibly reduced intestinal absorption. This is currently a challenge as almost all antiretroviral agents are available as oral preparations only.

A sensitivity analysis was performed (Figs A and B in S1 File). We re-evaluated our results by separating the studies into subgroups of ICU mortality and hospital mortality after ICU discharge; by excluding the largest study; by excluding the studies with lower methodologic quality (MINORS<10 points); and by excluding those with geographical and temporal variation.

There was still an observed lower short-term mortality associated with the use of HAART during the ICU stay, even after conducting the sensitivity analysis in regards to sample size, the study’s origin (high versus lower income countries), and the period of publication (before or after 2005 and 2010).

However, there was a difference of beneficial effect between the ICU and hospital mortalities subgroups: the use of HAART did not significantly impact the hospital mortality after the random effects analysis. This discrepancy might be explained by the fact that 3 [13,15,21] out of the five papers [13,15,17,20,21] which studied hospital mortality were outside the 95%CI region of the funnel plot. This suggests the possibility of either publication bias or a systematic difference between studies of higher and lower precision (typically ‘small study effects’) [12].

An assessment of symmetry in a funnel plot is typically subjective. Any evaluation is particularly challenging when the number of studies is small; funnel plots are considered unreliable methods of investigating potential bias if the number of studies is less than 10 [28]

Moreover, the heterogeneity analysis showed a substantial variability between studies. There was also high heterogeneity of methodological quality as expressed by the MINORS score. A meta-analysis of retrospective studies is expected to face the challenges of high heterogeneity and publication bias [29].

The patient population in different institutions and countries probably pertained unique characteristics and treatment preferences or protocols that influenced the spectrum of the disease. There were significant differences between hospital admission and discharge policies, as well as differences in practice patterns of the local staff [7]. These variables could have influenced the decision to start or not HAART during the ICU stay.

The articles presenting long-term outcomes disclosed very few ICU data. It was difficult to relate the critical and acute illness to a very long-term outcome. The long-term benefits of HAART are undeniable for the non-hospitalized patient, and well established on the literature [4,5,30–36]. As of now, there is no prospective study designed to assess the long-term effects of HAART in the survivors of critical illnesses.

This study has some limitations. Missing odds ratio values in some of the original articles made it unfeasible to calculate the meta-analysis using an adjusted odds ratio. Additionally, missing data and the small number of studies did not allow for a meta-analysis of the long-term outcomes. Clinical practice and demographics may differ across time, institutions, and countries. No randomized clinical trials were identified in the literature review; and observational retrospective studies may present heterogeneities, missing data, biases and confounders that could that have reduced the strength of our observations.

## Conclusions

The clinical implication of these findings is to support the initiation or maintenance of HAART in all HIV critically ill patients, as soon as the clinical condition dictates, even during an ICU stay. This meta-analysis suggests a promising trend for better outcomes in patients whom were treated with HAART during their ICU stay. Future randomized clinical trials are necessary to answer this question.

## Supporting information

S1 File PONE-D-17-21931R1-Supporting information. File with all the supporting information.
(DOCX)

S2 File PONE-D-17-21931R1-PRISMA checklist. The 27 checklist items pertain to the content of a systematic review and meta-analysis, which include the title, abstract, methods, results, discussion and funding.
(DOCX)
