# Supplementary material for: Highly active antiretroviral therapy for critically ill HIV patients: A systematic review and meta-analysis
Source: PLoS One. 2017 Oct 24;12(10):e0186968. doi: 10.1371/journal.pone.0186968 (PMC5655356; doi:10.1371/journal.pone.0186968)

**Highly active antiretroviral therapy for critically ill HIV patients: a systematic review and meta-analysis**

**Supporting information**

**Appendix: Methodological Index for Non-Randomized Studies (MINORS)**

**Criteria for non-comparative studies:**

1. An openly stated aim: the question addressed should be precise and relevant in light of the available literature

2. Inclusion of consecutive patients: all patients potentially fit for inclusion (satisfying the criteria for inclusion) have been included in the study during the study period (no exclusion or details about the reasons for exclusion)

3. Prospective collection of data: data were collected according to a protocol established before the beginning of the study

4. Endpoints appropriate to the aim of the study: an unambiguous explanation of the criteria used to evaluate the main outcome, which should be in accordance with the question addressed by the study. Additionally, the endpoints should be assessed on an intention-to-treat basis.

5. Unbiased assessment of the study endpoint: blind evaluation of objective endpoints and double-blind evaluation of subjective endpoints. Otherwise, the reasons for not blinding should be stated.

6. Follow-up period appropriate to the aim of the study: the follow-up should be sufficiently long enough to allow the assessment of the main endpoint and the possible adverse events.

7. Loss to follow-up of less than 5%: all patients should be included in the follow-up. Otherwise, the proportion lost to follow-up should not exceed the proportion experiencing the major endpoint.

8. Prospective calculation of the study size: information of the size of a detectable difference of interest with a calculation of 95% confidence interval, according to the expected incidence of the outcome event, and information about the level of statistical significance and estimates of power when comparing the outcomes.

**Additional criteria for comparative studies:**

9. An adequate control group: having a gold-standard diagnostic test or therapeutic intervention recognized as the optimal intervention according to the available published data.

10. Contemporary groups: control and studied group should be managed during the same period (no historical comparison).

11. Baseline equivalence of groups: the groups should be similar regarding the criteria other than the studied endpoints. Absence of confounding factors that could bias the interpretation of the results.

12. Adequate statistical analysis: whether the statistics were in accordance with the type of study with the calculation of confidence intervals or relative risk.

**Score system:** The items are scored 0 (not reported), 1 (reported but inadequate) or 2 (reported and adequate). The ideal global score was 16 for non-comparative studies and was 24 for comparative studies.

[**Slim K, Nini E, Forestier D, et al. (2003) Methodological index for non-randomized studies (MINORS): development and validation of a new instrument. ANZ J Surg 73:712–716.**](about:blank)

**Table A: Studies mortalities characteristics**

| **Study** | **HIV (n)** | **HAART (n)** | **Mortality n (%)** | **Univariate analysis** | **Multivariate analysis** |
| --- | --- | --- | --- | --- | --- |
| **ICU mortality** | | | | | |
| **Adlakha et al.** | 192 | 107 | total:43 (22.39%)  *HAART:* 14  *no HAART:* 29 | OR^a^ 1.87 (0.95-3.66),  p-value 0.068 | OR^a^ 2.24 (1.04-4.94), p-value 0.047 |
| **Amâncio et al.** | 125 | 19 | total: 58 (46.4%)  *HAART*: 3  *no HAART:* 43 | OR 0.26 (0.08 – 0.82), p-value <0.05 | OR 0.19 (0.05-0.77),  p-value <0.05 |
| **Cribbs et al.** | 165 | 58 | total: 58 (35%)  HAART: 27  no HAART: 48 | No regression data^b^ | No regression data^b^ |
| **Croda et al.** | 278 | 204 | total: 154 (55.4%)  *HAART:* 111  *no HAART:* 55 | OR 0.86 (0.50–1.47), p-value >0.2 | no data |
| **Meybeck et al.** | 91 | 32 | total: 17 (19%)  *HAART:* 5  *no HAART:* 12 | p-value 0.78^d^ | no data |
| **Morquin et al.** | 98 | 29 | total: 36 (36.7%)  *HAART:* 6  *no HAART:* 36 | OR 0.339 (0.123-0.937), p-value 0.037 | OR 0.278 (0.082-0.939), p-value 0.039 |
| **van Lelyveld et al.** | 80 | 29 | total: 25 (31%)  *HAART:* 10  *no HAART:* 15 | No regression data^b^ | No regression data^b^ |
| **Vargas - Infante et al.** | 90 | 35 | total: 52 (58.4%)  *HAART:* 10  *no HAART:* 42 | HR 0.25 (0.12-0.5), p-value <0.0001 | HR 0.3 (0.2-0.7), p-value 0.003 |
| **Hospital mortality after ICU discharge** | | | | | |
| **Adlakha et al.** | 192 | 107 | total: 58 (30%)  *HAART:* 21  *no HAART:* 38 | no regression data^b^ | no regression data^b^ |
| **Amâncio et al.** | 125 | 19 | total: 85 (68%)^a^ | no regression data^b^ | no regression data^b^ |
| **Barbier et al.** | 147 | 43 | total: 29 (19.7%)  *HAART:* 13  *no HAART:* 16 | no regression data^b^ | no regression data^b^ |
| **Chiang et al.** | 135 | 82 | total: 66 (48.9%)^a^ | OR 1.12 (0.56-2.24) p- value 0.7 | no data |
| **Cribbs et al.** | 165 | 58 | total: 82 (50%) | No regression data^b^ | No regression data^b^ |
| **Greenberg et al.** | 125 | 43 | total: 52 (42%)  *HAART:* 15  *no HAART:* 37 | no data | OR 0.61 (0.24-1.54), p-value 0.18 |
| **Morris et al.** | 58 | 12 | total: 32 (55.17%)  *HAART:* 3  *no HAART:* 29 | OR 0.2 (0.05-0.8), p-value 0.03 | OR 0.14 (0.02-0.84), p-value 0.03 |
| **van Lelyveld et al.** | 80 | 29 | total: 36 (45%)  *HAART:* 16  *no HAART:* 20 | no regression data^b^ | no regression data^b^ |
| **Long-term mortality after ICU discharge** | | | | | |
| **Amâncio et al.** | 125 | 19 | total 2 years: 38.8%^c^ | No regression data^b^ | No regression data^b^ |
| **Croda et al.** | 278 | 204 | total: 180 days: 193 (69.4%)  *HAART:* 139  *no HAART:* 54 | HR 0.5 (0.31-0.98), p-value<0.05 | HR 0.5 (0.35-0.71), p-value <0.05 |
| **Meybeck et al.** | 91 | 32 | total 180 days: 25 (69.4%)  *HAART:* 9  *no HAART:* 16 | p-value 0.99^d^ | log rank 0.04 |
| **Morquin et al.** | 98 | 29 | total 1 year: 61 (62.2%)^a^ | 0.807 (0.277-2.355), p-value 0.695 | HR 0.166 (0.043-0.642), p-value 0.009 |
| **van Lelyveld et al.** | 80 | 29 | total 1 year: 44 (52.94%)  *HAART:* 20  *no HAART:* 22 | OR 0.86 (0.99-8.29), p-value 0.05 | OR 1.12 (0.35-3.56), p-value 0.78 |
| **Vargas - Infante et al.** | 90 | 35 | total 90 days: 62 (69.6%)^a^ | HR 0.3 (0.2-0.6), p-value <0.0001 | HR 0.3 (0.2-0.7), p-value 0.002 |

*n* Absolute number; *HIV(n)* the number of HIV patients; *HAART(n)* the number of HIV patients who received highly active antiretroviral therapy (HAART) during Intensive Care Unit (ICU) stay; *OR* odds ratio; *HR* hazard ratio

^a^ OR calculated for survival

^b^ No regression data because Pearson's chi-square test with p-value > 0.05 showed no association to the outcome

^c^ No different mortality data for treated versus nontreated patients (HAART v. no HAART)

^d^ Only p-value data are available

**Fig A. Highly active antiretroviral therapy (HAART) effects on short-term mortality - sensitivity analysis**


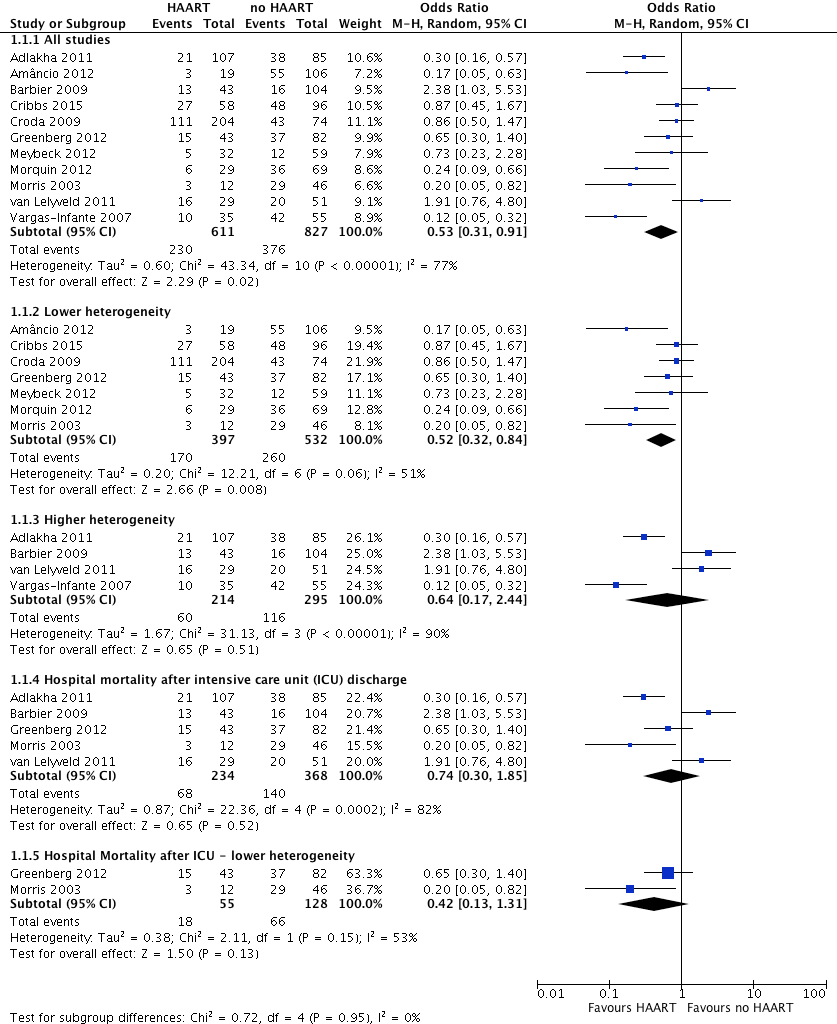


**
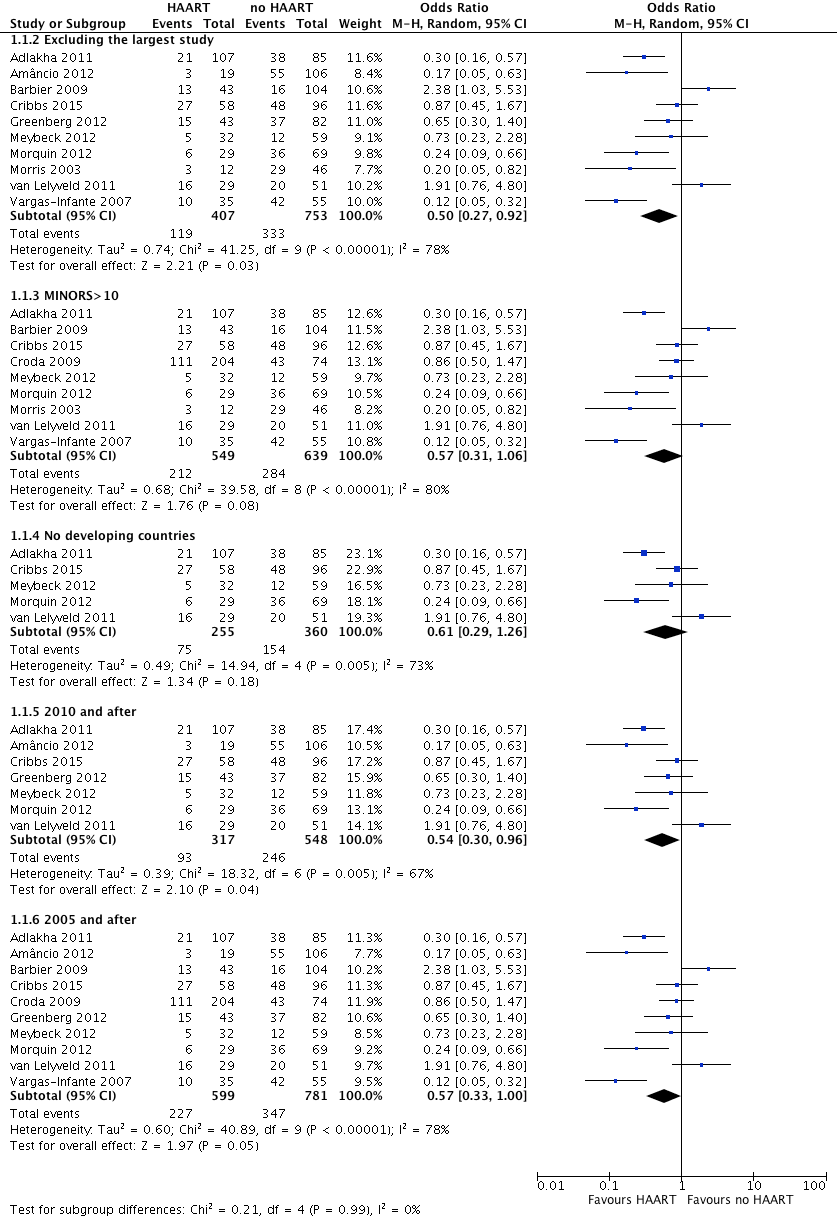
**

**Fig B - Intensive care unit mortality**


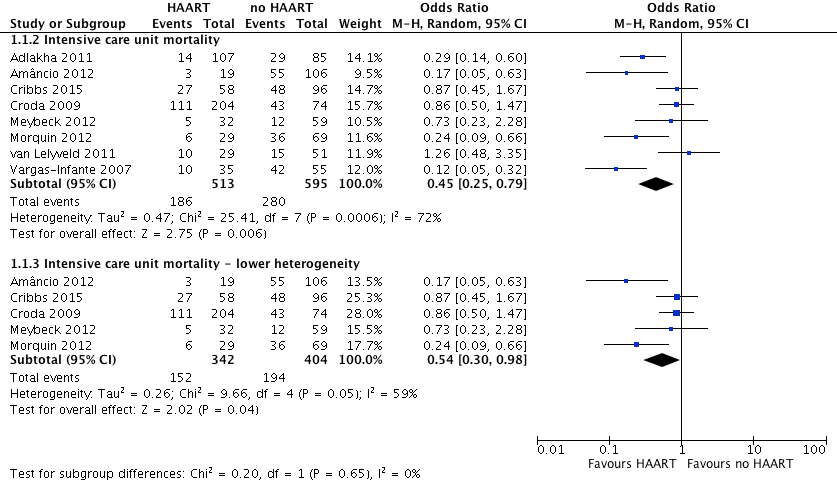

Supplement: S1 File — File with all the supporting information. (DOCX) [file pone.0186968.s001.docx]
